# Trephining; Its Indications, Technique, After-Treatment and Sequelæ

**Published:** 1899-07

**Authors:** L. A. Merillat


### DEPARTMENT OF SURGERY.

BY L. A. MERILLAT, V.S.,
SECRETARY OF M'KILLIP VETERINARY COLLEGE, CHICAGO.

ASSISTED BY

W. E. A. WYMAN, M.D.V., V.S., MILWAUKEE, WIS.

J. A. SLOAN, B.SC., M.D.V., SAN FRANCISCO, CAL.

DR. ROBERT JAY, M.D.V., WEST LIBERTY, IA.

### TREPHINING; ITS INDICATIONS, TECHNIQUE, AFTER-TREATMENT, AND SEQUELÆ. (Concluded from page 299).

TECHNIQUE. The following surgical lines are useful for locating internal structures of the head, and will be referred to hereafter in this article.

*A*. A straight line extending from the *nasal canthus* to the *maxillo-nasal*[1] *notch.* This line passes over the maxillo-nasal suture and marks the division between the sinuses and nasal cavity anteriorly, and between the frontal and maxillary sinuses posteriorly.

*B*. A line passing over the nasal arch so as to connect the *middle of the maxillary spines.* This line traverses the maxillo-malar suture, and intersects line *A* at a point where the frontal sinus is usually trephined.

*C*. A line passing over the nasal arch connecting the *anterior extremities of the maxillary spines.* This line passes over, or just behind, the infraorbital foramen which is found exactly midway between the extremity of the spine and line *A*.

*D*. A line connecting the *nasal canthi.* This line marks the union of the frontal and nasal bones, as well as the division between the nasal and cranial cavities, for all practical purposes.

It matters not for what purpose trephining is performed the technique need vary but little so far as the actual trephining is concerned. The location of the openings, and the subsequent steps after the skull-plate is removed, alone, vary according to the indication.

*Instruments, etc.* Scalpel, dissecting-forceps, artery-forceps or

[1] This name is adopted for the notch intervening between the base of the nasal peak and the upper branch of the premaxilla.

thumb-snaps, trephines (assorted), bone-chisels, periosteotome, needle and sutures, long-piped syringes, sponges, antiseptics in abundance, wadding of oakum or cotton.

*Restraining Horses for Trephining.* With the assistance of cocaine anæsthesia ordinary trephining can be performed in the standing posture, but when a tooth is to be removed, a tumor is to be ablated, or in very nervous animals, the recumbent is not only preferable but essential to insure success. It is a grave error in this as in many other surgical operations to operate in the standing posture. Surgery within the sinuses or nasal cavities requires careful manipulation and examination within to make sure that nothing is left undone, that no recesses are left uncleansed, and that no necrosed tissue, foreign bodies, or portions of tumors are permitted to remain and thereby perpetuate the disease at hand. For these reasons, as well as the safety of the operator, the recumbent position is essential in a large majority of cases.

Chloroform anæsthesia is not always necessary, and in fact is harmful in some instances. It is harmful when there is profuse hemorrhage, or when considerable irrigating fluid is necessary to cleanse the cavities. While anæsthetized, the blood or solution so used may readily find its way into the lower air-passages and prove a cause of deglutition pneumonia. It is therefore advisable to omit the general anæsthetic when possible, in fact in most cases; when used, the poll must be kept highly elevated so that fluids will find their way to the anterior nares.

*Standing Posture.* This method is recommended only for the simplest trephining operations. It must never be adopted for punching out teeth, nor for the removal of nasal tumors. Teeth can, of course, be punched out in this position, but such operations are seldom satisfactory and cannot be performed as accurately as a skilful surgeon desires.

The seat of operation is anæsthetized with cocaine, the horse backed into a narrow single stall, the affected side exposed by tying the head nearest to the opposite post, a good twitch is applied, and the operation may then be commenced some twenty minutes after the cocaine injection. The Lucas dental halter is very useful for the purpose, as the head can then be elevated or lowered to the most advantageous position.

*Recumbent Position.* This position is selected for all major trephining operations, in order to insure thoroughness of the procedure. An operating table is very useful, as the head can be more firmly secured then with other casting appliances. The body must be firmly fastened to preclude any antero-posterior movement, a folded blanket is arranged under the poll to elevate it, a small rope is looped around the neck of the inferior maxilla, passed through a hole in the table and held firmly by an assistant, who also applies his weight to the poll. The head should be slightly but not forcibly extended. Excessive extension of the head prevents normal deglutition and would encourage the passage of fluids into the trachea. In this position every detail of the operation can be accurately and thoroughly executed from the very first to the very last step.

In the absence of a table the aim should be to place the head in the same position, but on account of the animal's struggles the operation cannot be as methodically performed. The head is slightly elevated at the poll, as on the operating table, and is held as firmly as possibly by a strong assistant while another with a strong twitch attends to the muzzle.

*The Location of Openings.* The seat of operation must be selected with precise accuracy. More will depend upon this than any other part of the operation. The rule should be to select the most advantageous point for the particular operation at hand, and then deviate from it only when important vessels, nerves, or ducts would have to be sacrificed. The structures which should never be wounded are:

*a.* Steno's duct.
*b.* The glosso-facial artery and vein.
*c.* The lachrymal conduit.
*d.* The superior dental canal and contents.
*e.* The inferior dental canal and contents.
*f.* The superior maxillary division of the trifacial near its exit from the infraorbital foramen.

An example of this is found in removing the third superior molar in young horses. Its fang lies immediately beneath the infraorbital foramen containing the whole division of the trifacial nerve. To make an opening in the most advantageous location for its removal would result in total destruction of this nerve. The same condition is found in removing the fourth inferior molar which could be more easily extracted were it not for Steno's duct and the submaxillary artery.

*To remove a tooth* the opening must be so situated that the punch can be placed on a line with its long axis. The location will vary with the age of the patient. In young horses, the fangs of the molars being long, the opening must of necessity be made nearer the median line of the head, while in adult and aged horses they must be made nearer and nearer to the maxillary spine, as the length of the fang, indicated by the age, would demand.

The point is selected after the animal is secured and otherwise prepared for operation. With the aid of a speculum the hand is placed into the mouth and the finger pressed against the cheek opposite the offending member, and from the bulge thus made in the cheek externally a measurement is made upward to a point indicated by the animal's age.

But in no instance should the opening be beyond the maxillo-nasal suture nor too near the maxillary spine. The range made necessary by age is from the maxillo-nasal suture to a line two-and-a-half centimetres from the border of the spine. This is a point that cannot be too often repeated and must, to insure the greatest success, always be followed. It should only require a hint to elucidate the fact that if the punch is not on a direct line with the tooth it will require very forcible blows to dislodge it and thus damage the bones and surrounding structures. Often the damage is much worse than the original trouble.

The fangs of the third, fourth, and fifth inferior molars have a marked backward direction, and therefore the openings to remove them must be made accordingly,—for the fourth at the border of the jaw just behind the glosso-facial artery, and for the third just in front of this vessel. These are the only teeth that necessitate a modification of the above method.

*To empty, drain, and irrigate the sinuses.* For this purpose the opening must be located so as to allow free exit to the discharges and at the same time give access to every recess of the cavities for irrigation. To accomplish this two openings are necessary, one for the frontal and one for the maxillary sinus. The one for the frontal sinus is located in the postero-internal angle formed by the intersection of lines A and B. The opening is not made at the intersection but in the angle as above stated. In the average-sized horse this point will be found about six centimetres in an antero-internal direction from the nasal canthus. This does not open the lowest point of the frontal sinus, but is as low as can be made and still give access to the upper recesses.

For the maxillary sinus select a point near the anterior extremity of the spine, and about two centimetres from its base. Openings made for the removal of the fourth or fifth molars will also answer, but when no tooth is removed the above point should be selected. The small or anterior maxillary sinus is also exposed by this opening.

*For fractures* the location must vary in each case. Often the removal of a loose segment is all that is necessary, as the only aim is to admit the finger to replace the displaced section. Whenever this is not found advisable an opening is made adjacent to the region, below it if possible.

*For Turnsickness in Sheep.* The point selected for this operation is determined as follows: A point one-and-a-half to two centimetres from the centre of the horn (horn rudiment in females) and five millimetres from the median line. The affected side is determined by the movements of the animal, as it always moves toward the affected side.

*For the Ablation of Nasal Tumors.* Several openings are usually necessary. They must always be located upon the nasal bone and must vary in location according to the location of the tumor, but never external to line A. The antero-posterior range is over the whole course of the nasal bone. The exact location of nasal tumors can seldom be determined before the operation. It is, therefore, advisable in each case to begin making openings at the very edge of the nostril and then at regular intervals backward until the whole tumefaction is exposed. The first one is usually made through soft tissues only, from the border of the nostril (but not through the border) to the maxillo-nasal notch. This will admit the finger a considerable distance up the nasal cavity and will assist in diagnosing the location and extent of the tumor. Often they can be removed from this opening alone. If this is not possible, a second or even fourth opening is made. The openings through the nasal bone should be rectangular, and at least one-and-a-half centimetres intervening between them to insure prompt regeneration of the nasal roof.

(To be continued.)

L. A. Merillat, V.S.

